# Experimental evolution alters the rate and temporal pattern of population growth in *Batrachochytrium dendrobatidis*, a lethal fungal pathogen of amphibians

**DOI:** 10.1002/ece3.1199

**Published:** 2014-09-03

**Authors:** Jamie Voyles, Leah R Johnson, Cheryl J Briggs, Scott D Cashins, Ross A Alford, Lee Berger, Lee F Skerratt, Rick Speare, Erica Bree Rosenblum

**Affiliations:** 1 Department of Biology, New Mexico TechSocorro, New Mexico, 87801; 2 Department of Integrative Biology, University of South FloridaTampa, Florida, 33620; 3 Department of Ecology, Evolution and Marine Biology, University of CaliforniaSanta Barbara, California, 93106; 4 School of Public Health, Tropical Medicine and Rehabilitation Sciences, Amphibian Disease Ecology Group, James Cook UniversityTownsville, Queensland, 4811, Australia; 5 School of Marine and Tropical Biology, Amphibian Disease Ecology Group, James Cook UniversityTownsville, Queensland, 4811, Australia; 6 Department of Environmental Science, Policy and Management, University of California- BerkeleyBerkeley, California, 94720-3144

**Keywords:** Amphibian chytridiomycosis, amphibian declines, *B*a*trachochytrium dendrobatidis*, evolution of virulence, experimental evolution, host–pathogen interactions, serial passage experiments

## Abstract

Virulence of infectious pathogens can be unstable and evolve rapidly depending on the evolutionary dynamics of the organism. Experimental evolution can be used to characterize pathogen evolution, often with the underlying objective of understanding evolution of virulence. We used experimental evolution techniques (serial transfer experiments) to investigate differential growth and virulence of *B*a*trachochytrium dendrobatidis* (*Bd*), a fungal pathogen that causes amphibian chytridiomycosis. We tested two lineages of *Bd* that were derived from a single cryo-archived isolate; one lineage (P10) was passaged 10 times, whereas the second lineage (P50) was passaged 50 times. We quantified time to zoospore release, maximum zoospore densities, and timing of zoospore activity and then modeled population growth rates. We also conducted exposure experiments with a susceptible amphibian species, the common green tree frog (*Litoria caerulea*) to test the differential pathogenicity. We found that the P50 lineage had shorter time to zoospore production (*T*_*min*_), faster rate of sporangia death (*d*_*s*_), and an overall greater intrinsic population growth rate (*λ*). These patterns of population growth *in vitro* corresponded with higher prevalence and intensities of infection in exposed *Litoria caerulea*, although the differences were not significant. Our results corroborate studies that suggest that *Bd* may be able to evolve relatively rapidly. Our findings also challenge the general assumption that pathogens will always attenuate in culture because shifts in *Bd* virulence may depend on laboratory culturing practices. These findings have practical implications for the laboratory maintenance of *Bd* isolates and underscore the importance of understanding the evolution of virulence in amphibian chytridiomycosis.

## Introduction

Understanding pathogen biology, life-history strategies and evolutionary dynamics is important in multiple medical and bioscientific fields (Stearns and Koella 2008). The study of pathogens and the evolution of virulence is not only critical for wildlife and human health at the individual level, but also at a much larger scale because infectious diseases can alter host population densities, community dynamics, and potentially entire ecosystems (Scott 1988; De Castro and Bolker 2005; Whiles et al. 2006). Furthermore, insights into pathogen evolution provide a better understanding of the mechanisms driving disease dynamics and allow for improved predictions of disease spread and potential impacts (Bull 1994; Ebert and Bull 2003). Until relatively recently, however, the factors influencing pathogen evolution were difficult to study and therefore not well understood (Bull 1994; Ebert and Bull 2003).

Historically, the application of experimental evolution with infectious agents (studies in which virulence is artificially modified) was a turning point in the study of infectious disease (Bezin 2003). Louise Pasteur first employed serial passage techniques in the 18th century (Bezin 2003), and since then, serial passage experiments (SPEs) have become a cornerstone in the study of infectious disease and the development of vaccines (Bezin 2003). SPEs have been used for *in vitro* and *in vivo* investigations of a wide variety of pathogens: viruses (Schlesinger et al. 1956; Beare et al. 1968), bacteria (Cushion and Walzer 1984; Maisnier-Patin et al. 2002; Somerville et al. 2002), protozoa (Diffley et al. 1987), and fungi (da Silva Ferreira et al. 2004; Wang et al. 2008). Thus, evolutionary principles and the notion that pathogens should adapt to novel environments have long been applied in the study of infectious disease, even before we had a complete understanding of how or why evolutionary shifts in pathogen virulence might occur (Bezin 2003; Hanley 2011).

During experimental evolution using serial passage experiments (SPEs), pathogens are transferred to alternative hosts or to new artificial environments at a specific point in the pathogen life cycle (Ebert 1998; Ford et al. 2002). The advantage of SPEs is that alterations in pathogen genotype, phenotype, and virulence can be tracked in real time (Ebert 1998). Many SPEs studies have demonstrated that pathogens adapt to novel environments (e.g., in culture or alternative hosts) relatively rapidly (reviewed in Ebert 1998) and that they frequently become less efficient at surviving in their natural hosts (Ebert 1998; Ford et al. 2002). More specifically, SPEs can lead to shifts in pathogen reproductive rates (a common component of virulence) such that pathogen replication is attenuated (Ebert 1998; Hanley 2011). However, shifts in virulence are not obligately unidirectional toward hypovirulence (Ebert 1998). Pathogens can also exhibit shifts toward hypervirulence or revert from a hypovirulent state (Mastroeni et al. 2011). Some studies have shown that shifts in pathogen virulence will greatly depend on passage practices. For example, the timing of passage (i.e., the point of the pathogen’s life cycle at which it is propagated) and the conditions of propagation (e.g., nutrient and thermal conditions) can influence the direction of shifts in pathogens’ abilities to exploit the available resources (Ford et al. 2002).

We used experimental evolution to investigate growth and virulence (i.e., pathogenicity) of a fungal pathogen of amphibians. *Batrachochytrium dendrobatidis* (hereafter “*Bd*”) is an aquatic fungus causes the disease chytridiomycosis and is highly virulent to many species of amphibians (Berger et al. 1998, 2005; Voyles et al. 2009; Alford 2010). *Bd* infects epidermal cells and causes disruption of electrolyte (e.g., sodium) transport across the epidermis, and subsequent cardiac arrest (Voyles et al. 2009). Although the mechanisms by which *Bd* disrupts epidermal function are not yet fully understood, potential virulence factors have been identified (Rosenblum et al. 2009; Fites et al. 2013). In addition to virulence factors, *Bd* reproductive rates play an important role in pathogenesis and disease development, contributing to intensity of infection and thus *Bd* load in an individual host (Voyles et al. 2009; Vredenburg et al. 2010). Although we now have a much better understanding of chytridiomycosis pathophysiology, host immunity, and disease ecology (reviewed in Kilpatrick et al. 2010; Venesky et al. 2013), researchers have only relatively recently turned their attention to investigations on differential virulence among *Bd* isolates, evolutionary shifts in *Bd*, and host–pathogen coevolution (Fisher et al. 2009; Farrer et al. 2011, 2013; Voyles et al. 2012; Rosenblum et al. 2013).

Several lines of evidence suggest that evolution in *Bd* virulence may be occurring quite rapidly. First, although chytridiomycosis has been responsible for many catastrophic amphibian declines and even local extinction events, some species and populations of species have survived initial declines and now persist in the wild with *Bd* infections (Retallick et al. 2004; Woodhams and Alford 2005; Alford 2010; Puschendorf et al. 2011). One investigation that explicitly focused on the patterns of disease emergence and host declines has suggested that evolution of *Bd* virulence must be occurring across time and space (Phillips and Puschendorf 2013). Second, laboratory studies have documented *Bd* attenuation with successive *in vitro* propagation using routine culture maintenance practices (Brem et al. 2013; Langhammer et al. 2013). In these studies, *Bd* attenuation was characterized by reductions in zoospore production rates (i.e., pathogen replication) and in pathogenicity in live amphibian inoculation experiments (Brem et al. 2013; Langhammer et al. 2013). Third, recent molecular studies that have examined the genomic diversity of *Bd* isolates (i.e., isolates collected from widespread geographic and multiple host species origins) have suggested that *Bd* has a highly dynamic genome with multiple possible mechanisms that could be contributing to a complex evolutionary history (Farrer et al. 2013; Rosenblum et al. 2013). Taken together, these three lines of evidence suggest that further investigations focused on evolution in *Bd* may help resolve how this pathogen has been so successful to such a broad range of host species in a wide variety of environments.

We aimed to understand how *Bd* growth and virulence are altered with experimental evolution using serial passage experiments. We used a single isolate of *Bd* and derived two lineages that were treated identically except for the length of time they were propagated in artificial media (i.e., 10 vs. 50 passages). By passaging *Bd* at the peak of zoospore production, our serial propagation imposed artificial selection for an early release of zoospores and high densities of zoospores. We found that the differences in passage histories translated into changes in *Bd* population growth rates *in vitro* and *Bd* growth and virulence *in vivo*.

## Methods

### Serial transfer experiments

We originally obtained the isolate, GibboRiver-L.lesueuri-00-LB-1, from a diseased juvenile *L. lesueuri* that was collected in the wild and died in captivity. This isolate was cultured on tryptone/gelatin hydrolysate/lactose (TGhL) agar with antibiotics (Longcore et al. 1999) and then cryo-archived according to standard protocols (Boyle et al. 2003). We revived one aliquot of the cryoarchived culture (Boyle et al. 2003) and passaged it into liquid TGhL broth in 25-cm^2^ cell culture flasks (Longcore et al. 1999). We incubated the cultures at 22°C and inspected the flasks daily to monitor zoospore encystment and maturation of the zoosporangia. We passaged cultures into new media when zoospore density peaked (~5–7 days based on previous experiments; Voyles 2011; Voyles et al. 2012) and repeated this procedure for 50 passages. This *Bd* lineage with a higher number of passages will be referred to as “P50”. We revived a second aliquot of the same isolate 250 days later and treated the *Bd* culture identically for 10 passages. This lineage with a lower number of passages will be referred to as “P10”. Thus, the two lineages were maintained in identical conditions (i.e., in the same laboratory, using identical techniques and equipment, maintained at the same temperature and by the same investigator) except that one was passaged 50 times and one was passaged 10 times. This approach allowed us to test the two lineages simultaneously in a common garden experiment.

We filtered the two *Bd* lineages through sterile filter paper (Whatman, 3) to remove sporangia. We washed zoospores using a gentle centrifugation (500 *g* for 10 min), removing the supernatant, and resuspending the zoospores in fresh TGhL. We determined the zoospore concentrations using a hemocytometer (Improved Neubauer Bright-line) and adjusted to 90 × 10^4^ zoospores mL^−1^ as needed by adding TGhL. We conducted the phenotyping experiments in sterile 96-well plates (Tissue culture test plates-96, TPP, company info). We pipetted the zoospore inocula (50 *μ*L) into each of 20 wells containing 50 *μ*L TGhL media. The plate had a perimeter of 36 wells with 100 *μ*L sterile water to avoid evaporation. We inspected the plates daily to monitor zoospore encystment, development, and maturation of the zoosporangia. Once the maturing zoosporangia produced the first zoospores, we quantified zoospore density daily by randomly selecting 10 wells of each of the two lineages, drawing off 30 *μ*L of supernatant, and counting zoospore numbers using a hemocytometer.

### Model development

To understand differences between the two lineages, we used a mathematical model to analyze our empirical data. Specifically, we used a delay differential equation model that was developed for a previous study on *Bd* (Voyles et al. 2012). The model used the data on the concentration of zoospores produced in the next generation from the initial cohort of zoospores placed in each well of the 96-well plate and follows the dynamics of: C(t) = the concentration of the initial cohort of zoospores; S(t) = the concentration of zoospore-producing sporangia; and Z(t) = the concentration of zoospores in the next generation. The initial cohort of zoospores, C(t), started at a concentration of 90 × 10^4^ zoospores per mL, and zoospores in this initial cohort settle and become sporangia at rate *s*_*r*_ or die at rate *μ*_*z*_. *f*_*s*_ is the fraction of sporangia that survive to the zoospore-producing stage.

The model assumed that it takes a minimum of *T*_*min*_ days before the sporangia produce zoospores, after which they produce zoospores at rate *η*. Zoospore-producing sporangia die at rate *d*_*s*_. The concentration of zoospores, Z(t), is the state variable actually measured in the experiments, and it is assumed that these zoospores settle (*s*_*r*_) or die (*μ*_*z*_) at the same rates as the initial cohort of zoospores. The equations that describe this are as follows:

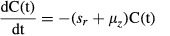
(1)

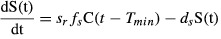
(2)

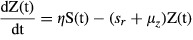
(3)

Zoospore-producing sporangia die at rate *d*_*s*_. For this model, the population growth rate *λ* can be calculated numerically from the transcendental equation:

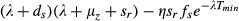


We used a Bayesian approach to infer the values of the model parameters in Equations (1)–(3) that best fit the experimental data from each *Bd* lineage. Our data consist of observations on the numbers of zoospores produced after serial passage treatments (i.e., on Z(t)), although we know the initial conditions of all the states. With discrete numbers of zoospores, we modeled our observations of the system at a set of discrete times *t*′ as independent Poisson random variables with a mean given by the solution of Equations 1 - 3, at times *t*′:

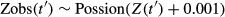


We tested for significant differences between the *Bd* lineages by fitting the model to the data from the two lineages combined (i.e., assuming both lineages had the same parameters) and comparing this to the fit of the model to each lineage separately (i.e., assuming different parameters for the two lineages) using the deviance information criterion (DIC).

### Experimental inoculations

**Figure 1:**
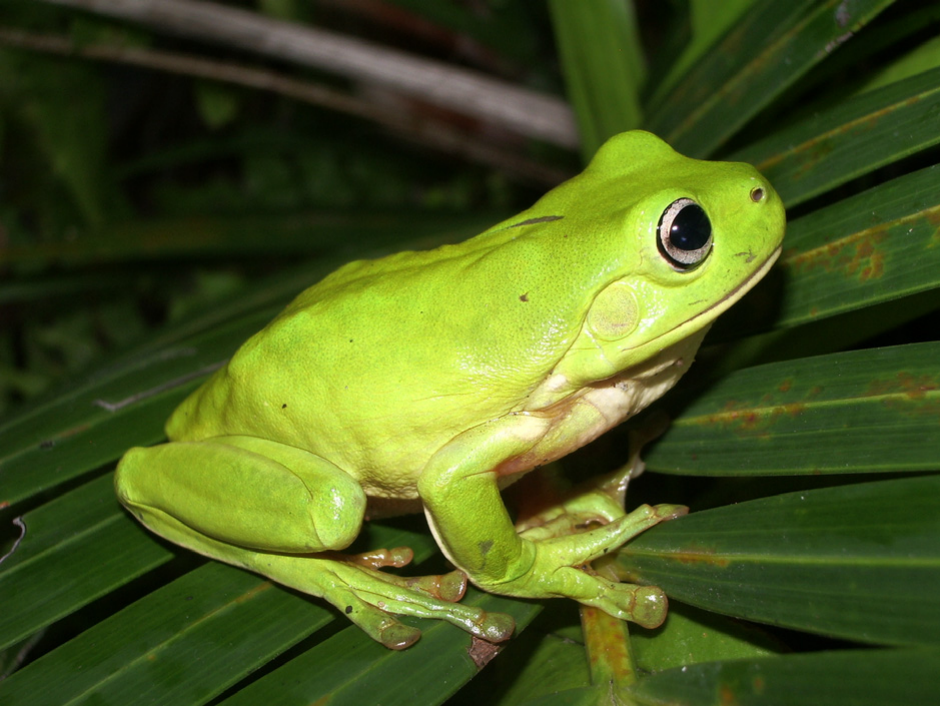
The common green tree frog (*Litoria caerulea*).

We collected adult common green tree frogs (*Litoria caerulea*; *N* = 30, mean mass: 21.34 ± 5.64 SD; Fig. 1) in January and February 2008 from residential areas of Townsville, Queensland, an area that is predicted to be unsuitable for *Bd* (Murray et al. 2011). We collected each animal using a new plastic bag and then transferred each individual to a plastic container (200 × 240 × 330 mm^3^), containing 250 mL of tap water. We maintained the containers in temperature- (18–23°C) and light (12L/12D)-controlled facilities at James Cook University, Townsville, Australia. We fed frogs vitamin-dusted crickets (medium-sized, Pisces Inc. Boulder, Colorado, USA) *ad libitum* twice per week. We also changed the tap water (250 mL) twice a week until experimental exposures began, and then, we replaced tap water with 20% Holtfretter’s solution (in mMol: NaCl (6.0), KCL (0.06), CaCl_2_ (0.09), NaCO_3_ (0.24), pH 6.5, 250 mL). We maintained the frog containers in a level position, so water covered the bottom, but frogs were able to climb up the dry walls.

To confirm that frogs were not infected with *Bd* prior to inoculation, we swabbed their ventral surfaces and digits and tested for *Bd* using a Taqman real-time polymerase chain reaction (PCR) assay (Boyle et al. 2004; Hyatt et al. 2007). More specifically, we swabbed the abdomen and ingroinal regions 10 times and each of the digits five times. For the PCR assay, we analyzed all samples in triplicate and compared them with Australian Animal Health Laboratory zoospore standards to determine zoospore equivalents (Hyatt et al. 2007).

For animal inoculations, we filtered *Bd* zoospores from the P10 and P50 lineages to remove sporangia (described above). We determined zoospore concentrations using a hemocytometer (Improved Neubauer Bright-line) and adjusted the zoospore concentrations to 93 × 10^4^ zoospores mL^−1^ by diluting with fresh TGhL media. We randomly assigned frogs to one of three treatment groups: two exposure groups (P10 and P50 isolate treatments) or a control group (*N* = 10 frogs per group). We inoculated frogs by exposure to *Bd* via shallow immersion in a bath of Holtfretter’s solution and sterile TGhL with *Bd* zoospores. We immersed the control frogs in a bath that contained Holtfretter’s solution and sterile TGhL with no *Bd* zoospores. After 24 h, we moved the frogs to fresh containers with 20% Holtfretter’s solution (pH 6.5). Following exposure to *Bd*, we collected skin swabs again at 17, 57, 104, 127, and 157 days postexposure. We did not collect mass during the course of the experiment to minimize disturbance to the animals, but we calculated the change in mass (final minus initial mass) by collecting mass at the beginning of the experiment and at the termination of the experiment.

## Results

**Figure 2:**
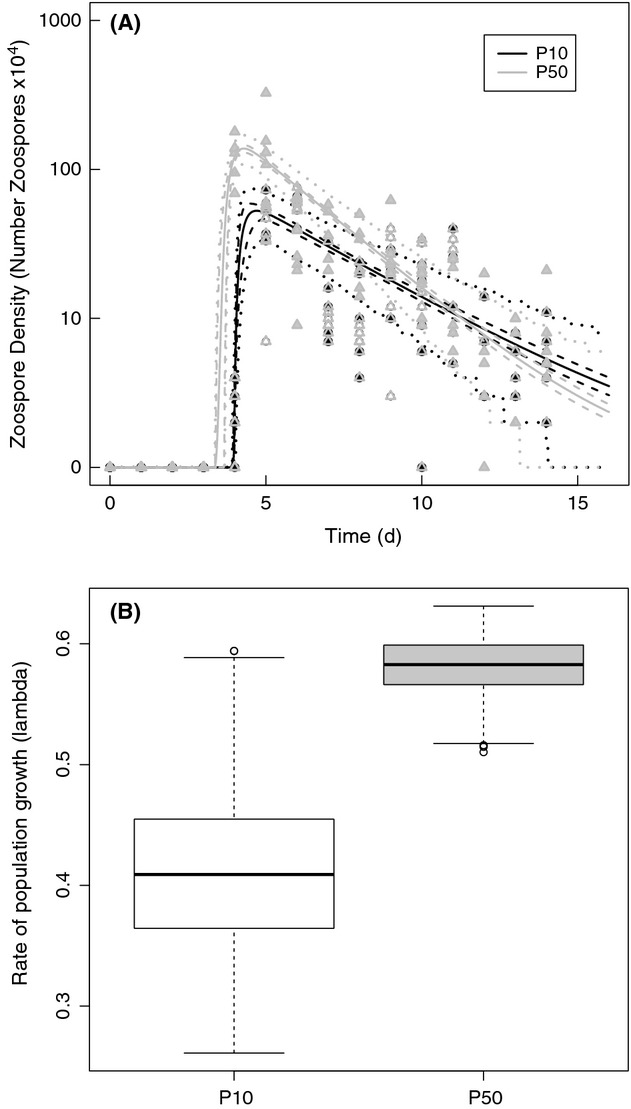
Line graph over time (A) and box-and-whiskers plots (B) showing modeled population growth rate (*λ*, lambda) of two lineages of *Batrachochytrium dendrobatidis*, GibboRiver-L.lesueuri-00-LB, that were serially passaged 50 times (P50; gray) and 10 times (P10; black).

**Figure 3:**
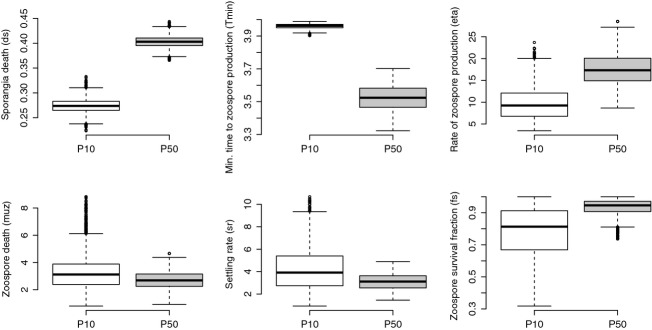
Box-and-whiskers plots for growth parameters for two lineages of *Batrachochytrium dendrobatidis* that were propagated for 50 passages (P50) or 10 passages (P10). Horizontal bars are medians and reflect lineage growth through time.

We found differences in population growth rate (lambda, *λ*) between the P10 and P50 lineages of *Bd* (Fig. 2A, B). We generated the 95% credible intervals for population growth rate using the posterior samples of the model parameters. The parameters that most likely contributed to these population differences include time to zoospore production (*T*_*min*_) and rate of sporangia death (*d*_*s*_) (Fig. 3). Although additional parameters such as rate of zoospore production (*η*) and fraction of zoospores that survive (*f*_*s*_) may have also contributed to our observed differences in lambda (*λ*), our model fitting indicates that these parameters are less likely to be different between the two lineages (Fig. 3).

For the P50 lineage, we found that the 95% credible intervals of the posterior distribution for time to zoospore production (*T*_*min*_) were lower and had no overlap with those for the P10 lineage (P10: *T*_*min*_ = 3.93–3.99; P50: *T*_*min*_ = 3.38–3.67; Fig. 3). Similarly, the intervals of the posterior distribution of sporangia death (*d*_*s*_) were higher in the P50 lineage compared with the P10 lineage (P10: *d*_*s*_ = 0.248–0.304; P50: *d*_*s*_ = 0.379–0.423; Fig. 3). As such, differences between the two lineages in the time to zoospore production and sporangia death most likely contributed to the net effects on population growth rate. The evidence for a higher population growth rate in the P50 lineage is further supported by the deviance information criterion (DIC) values, which indicate a considerably better fit for the two data sets fits with different parameters (DIC = 1753.318) compared with a single set of parameters (DIC = 2861.492). Thus, our model with separate parameters for the two populations explains the data much better than a model where both populations share the same parameters.

### Experimental inoculations of *Litoria caerulea*

**Figure 4:**
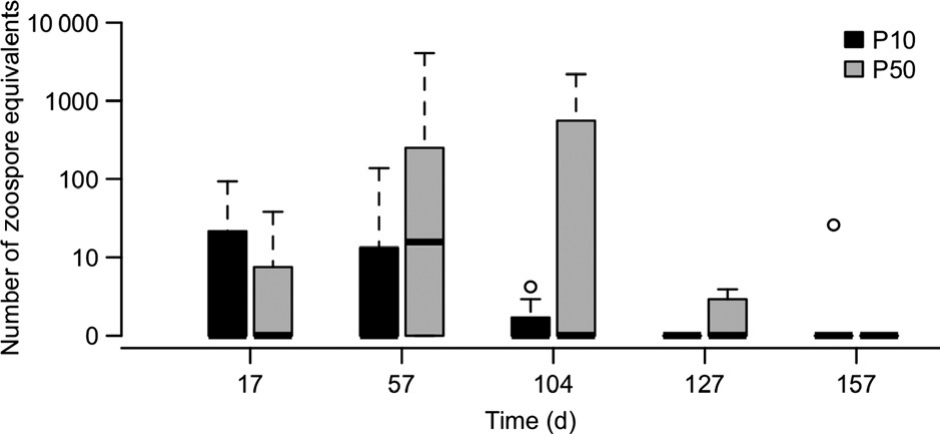
Intensity of infection in common green tree frogs (*Litoria caerulea*) that were infected with one isolate of *Batrachochytrium dendrobatidis* with two passage histories.

**Figure 5:**
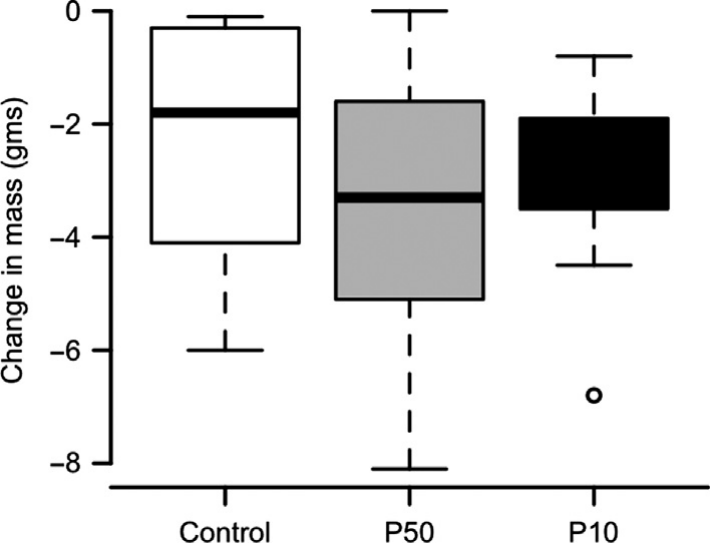
Change in mass (final weight minus initial weight) in *Litoria caerulea* experimentally exposed to two lineages of *Batrachochytrium dendrobatidis* (50 times (P50; gray) and 10 times (P10; black) or to a control solution (white bar)).

Although the results of our *in vitro* experiment and mathematical modeling indicated that the SPEs led to a higher population growth rate in the P50 culture, it was unknown whether this would translate to an increased growth rate, and hence virulence, in susceptible frogs. In our frog inoculation experiments, we used several response variables to assess differences in growth and virulence: infection prevalence and intensity (i.e., pathogen load), changes in mass, clinical signs of disease and mortality. Using PCR analysis on swab samples, we found that prevalence did not differ significantly (Fisher’s exact test, *P* = 0.63) between treatments. Eighty percent (8/10) *L. caerulea* exposed to P50 zoospores became infected during the experiment, while 60% (6/10) of frogs exposed to P10 zoospores became infected. The frogs that became infected by P50 zoospores had slightly higher intensities of infection than the frogs exposed to P10 zoospores, but this difference was not significant (repeated-measures ANOVA, *P* = 0.156; Fig. 4). The decrease in mass (final minus initial weight) was greatest in the P50 group. However, this change did not differ significantly from the P10 and control groups (ANOVA, *P* = 0.48; Fig. 5).

We observed mild clinical signs of infection including lethargy, inappetence, and slight skin discoloration in two frogs in the P50 group and two frogs in the P10 group. More severe clinical signs of infection (as per Voyles et al. 2009) did not develop in any group. Frogs with mild clinical signs seemed to recover, regaining normal color, activity, and appetite, by the termination of the experiment. Additionally, there was no mortality in any group.

## Discussion

Differential growth and virulence of *Batrachochytrium dendrobatidis* has been reported in multiple studies, but it is unclear why this phenotypic variation exists (Berger et al. 2005; Retallick and Miera 2007; Fisher et al. 2009; Voyles 2011). By reviving two aliquots of a single isolate of *Bd* and subjecting them to shorter or longer periods of passaging, we investigated selective effects of passage history on zoospore densities. Because we passaged the two lineages at the peak of zoospore densities, we eliminated the contribution of zoospores produced after this peak in each subsequent generation. This approach produced strong selection for early zoospore release and maximum zoospore densities. We predicted that 50 passages with this selective pressure would produce a greater response than 10 passages. We also expected that the P50 lineage, which produced more zoospores and had a higher population growth rate, might have been more pathogenic in our exposure experiments.

Our results suggest that passaging practices altered the rate and temporal pattern of zoospore production and population growth of Bd *in vitro*. We found that the time to zoospore production (*T*_*min*_) was faster and the overall population growth rate (*λ*) was higher in the P50 lineage (Fig 3). The changes in these parameters may reflect the culturing practice of passaging the *Bd* lineages at their first peak in zoospore production, thereby selecting for zoospores that are produced the earliest and make a substantial contribution to the overall population growth rate. These findings are important because they suggest that *Bd* evolves in culture and that more passages are likely to lead to greater divergence from the initial state of virulence in amphibian hosts. Additionally, the timing of propagation during *Bd*’s life cycle is appears to be critical; passaging at the height of zoospore production may also lead to faster population growth rates and higher virulence.

One possible limitation, however, is that the zoospore densities of the two lineages were not quantified immediately after revival or compared with the original strain (the “ancestor” strain) after serial passage treatments, so the differences we observed could conceivably have been caused by differences in the aliquots that were revived to start the P50 and P10 lineages. An additional objection could be made that the two lineages represent two separate evolutionary trajectories. Both of these objections are valid. However, both aliquots were samples that were originally derived from a single common source, and the P10 lineage remained frozen in liquid nitrogen until it was revived for this study. Furthermore, the two lineages were maintained in the same laboratory, using identical techniques and equipment, maintained at the same temperature and tested by the same investigator in multiple rigorous “common garden” experiments. Thus, our observations in the two lineages probably reflect true differences that resulted from laboratory practices at some point. We believe the most parsimonious explanation is that the differences were due to the serial culturing treatments.

One alternative experimental approach that could be used in future investigations is to test a single lineage that is subsampled and cryo-preserved at successive time points (e.g., Knies et al. 2006). We were unable to conduct this experiment, but we believe this design would allow researchers to further investigate the timing and conditions under which *Bd* changes during long-term serial passage treatments.

Overall, our results on the population growth rates in the P10 and P50 lineages suggest that patterns of zoospore production can vary depending on the timing of culturing practices. Quantifying parameters such as zoospore production in culture is valuable for understanding rates and patterns of population growth of *Bd in vitro*, but the implications for *Bd* growth *in vivo* and virulence are less clear. Because the P50 culture produced more zoospores, we predicted that it might be more virulent in inoculation experiments. This prediction was supported in our inoculation experiments by the patterns we observed in our measured response variables (i.e., higher prevalence and intensity of infection). However, these differences were not significant, and furthermore, there was no mortality in any group of *Litoria caerulea* exposed to P50 or P10 cultures. The lack of mortality was unexpected because this isolate, GibboRiver-L.lesueuri-00-LB with a similar passage history, was chosen due to its high level of virulence in parallel experiments (see Voyles et al. 2009). This isolate caused 90% mortality of *Litoria caerulea* in separate experiments using virtually identical methods, laboratory facilities, and equipment. Differences between the present study and the previously published experiments include the length of time frogs were held in captivity, the seasonal timing of animal exposures, and differences in isolate passage practices (cultures for the previous infection experiment had comparable passage history, but the lineages were maintained at 4°C rather than 22°C, which could be an important selective pressure; Voyles et al. 2012; Stevenson et al. 2013).

Our results challenge the general assumption that pathogens will attenuate in culture. It is commonly thought that as pathogens adapt to culture conditions, they lose their ability to exploit hosts as resources (Ebert 1998; Ford et al. 2002). Yet, evidence from several study systems suggests that shifts in virulence *in vitro* are not always unidirectional. Rather, passage timing (i.e., the point of the pathogen’s life cycle at which it is propagated) and the conditions of propagation (e.g., nutrient and thermal conditions) can influence shifts in virulence (da Silva & Sacks, 1987; Wozencraft and Blackwell 1987; Rey et al. 1990). Additionally, attenuated pathogens strains can rapidly revert to a virulent form when re-exposed to a naïve host (Cann et al. 1984; Macadam et al. 1989; Minor 1993; Nielsen et al. 2001). Thus, it is increasingly clear that virulence can be greatly affected by how pathogens are maintained and experimentally manipulated in the laboratory. We suggest that the timing of *Bd* propagation when maintaining cultures is critical because it may influence growth, zoospore production, and ultimately virulence of *Bd*.

Understanding how pathogens evolve *in vitro*, including attenuation and reversion to higher virulence, can advance our understanding evolution of pathogen virulence and has practical applications for disease research. The stability of virulence presents a considerable challenge for pathogen research (Michel and Garcia 2003), especially if virulence can evolve bi-directionally (Ebert and Bull 2003; Stearns and Koella 2008). For example, the ability to reliably infect hosts in controlled conditions is critical for the study of infectious disease (Ford et al. 2002; Brem et al. 2013). Culture history could also distort the outcome of experiments that are aimed at understanding host–pathogen interactions in nature (Langhammer et al. 2013). Additionally, studying changes in virulence represents an opportunity to pinpoint the mechanisms of pathogenesis. Culturing practices in which a pathogen is manipulated toward attenuation but then reverts to high virulence may reveal factors that determine the level of virulence for a particular host–pathogen dynamic. Thus, it is important to resolve how laboratory culture practices and experimental manipulation can influence pathogen growth, development, and virulence.
